# Spatial distribution of the Shannon entropy for mass spectrometry imaging

**DOI:** 10.1371/journal.pone.0283966

**Published:** 2023-04-06

**Authors:** Lili Xu, Kenji Kikushima, Shumpei Sato, Ariful Islam, Tomohito Sato, Shuhei Aramaki, Chi Zhang, Takumi Sakamoto, Fumihiro Eto, Yutaka Takahashi, Ikuko Yao, Manabu Machida, Tomoaki Kahyo, Mitsutoshi Setou

**Affiliations:** 1 Department of Cellular and Molecular Anatomy, Hamamatsu University School of Medicine, Hamamatsu, Shizuoka, Japan; 2 International Mass Imaging Center, Hamamatsu University School of Medicine, Hamamatsu, Shizuoka, Japan; 3 Graduate School of Medical Sciences Department of Integrative Anatomy, Nagoya City University, Nagoya, Aichi, Japan; 4 Department of Biomedical Sciences, School of Biological and Environmental Sciences, Kwansei Gakuin University, Sanda, Hyogo, Japan; 5 Department of Systems Molecular Anatomy, Institute for Medical Photonics Research, Preeminent Medical Photonics Education & Research Center Hamamatsu, Hamamatsu, Shizuoka, Japan; 6 JST, PRESTO, Kawaguchi, Saitama, Japan; Fisheries and Oceans Canada, CANADA

## Abstract

Mass spectrometry imaging (MSI) allows us to visualize the spatial distribution of molecular components in a sample. A large amount of mass spectrometry data comprehensively provides molecular distributions. In this study, we focus on the information in the obtained data and use the Shannon entropy as a quantity to analyze MSI data. By calculating the Shannon entropy at each pixel on a sample, the spatial distribution of the Shannon entropy is obtained from MSI data. We found that low-entropy pixels in entropy heat maps for kidneys of mice had different structures between two ages (3 months and 31 months). Such changes cannot be visualized by conventional imaging techniques. We further propose a method to find informative molecules. As a demonstration of the proposed scheme, we identified two molecules by setting a region of interest which contained low-entropy pixels and by exploring changes of peaks in the region.

## Introduction

Mass spectrometry imaging (MSI) enables us to acquire mass spectra and to visualize the spatial distribution of analytes simultaneously by ionizing them from the surface of a sample and identifying molecules [1–3]. The large amount of data gathered from MSI prompts us to explore physiological functions and pathological correlations.

Current progress in biological analyses enables instant access to massive amounts of data describing the detailed conditions of biological samples, initiating bioinformatics studies, and bringing great revolutions to biology and medicine [4]. The concept of the Shannon entropy has been applied to analyze these big data obtained from the biological samples. The Shannon entropy is commonly utilized in biology to measure diversity and defines how cells, genes, or molecules distribute and interact [5]. Initially, the Shannon entropy was utilized to represent the randomness of the DNA sequence composed of four nucleotides: adenine (A), cytosine (C), guanine (G) and thymine (T) [6, 7]. Since then, various kinds of entropy have been used on the genome: the metric entropy [8], the Renyi entropy [9], the diffusion entropy [10], and the topological entropy [11]. In systems biology, the amount of information is described by the Shannon entropy [12] and has been applied to examining the robustness of the signaling transmission through the different omics layers such as transcriptomics, proteomics, and metabolomics [13]. The Shannon entropy calculated from MSI data from peanuts was used to identify advanced glycation end products [14]. The use of the Shannon entropy for RNA-seq datasets was also proven to be useful for a quick and in-depth analysis of changes in the gene expressions [15]. Antibodies in human blood were detected by a peptide microarray, and the Shannon entropy calculated from the profile is proposed as an indicator of the health status of individuals and populations [16].

Initially, entropy was introduced in mass spectrometry as a tool to detect the pattern of a complex spectrum in extracting molecular information [17, 18]. Later, entropy was used to align high-resolution images [19] and to determine the spatial correspondences between the MSI data and the histological image for overlays [20]. See a book by Kaltashov and Eyles [21] and a review article by Aoyagi [22] about how entropy has been used in MSI. Moreover, the spectral binning was investigated with the Shannon entropy [23]. In [24], the Shannon entropy was used to match mass spectra to peptides and proteins. In [25], a data-targeted extraction method for metabolite annotation was proposed for liquid chromatography-high-resolution mass spectrometry.

Recently, entropy has started to be recognized as a physical quantity to be imaged not only a mathematical tool for MSI data processing. Aoyagi and her collaborators proposed a method based on the information entropy (Shannon entropy) for time-of-flight secondary ion mass spectrometry (TOF-SIMS) and showed that without peak identification the spatial distribution (heat maps) of the Shannon entropy of spectra indicates differences in materials and changes in the conditions of a material in a sample [26].

In this paper, we consider the information in the whole mass spectrum by observing the Shannon entropy and obtain the spatial distribution of the information by entropy heat maps for the matrix-assisted laser desorption/ionization (MALDI) MSI. In [27], mass spectral patterns were used for segmentation by deep learning but it was difficult to grasp the physical meaning of mass spectral patterns. In this study, we found that the spatial distribution of the Shannon entropy provides new images which contain information that cannot be obtained from images by optical microscopy nor conventional MSI. With entropy heat maps, we propose a method to select candidate peaks.

## Materials and methods

### Animals

For the mouse kidneys, C57BL/6JJmsSlc male mice of 3- and 31-month-old were used in the experiment. All mice were born in our animal facility and their parents were purchased from SLC (Hamamatsu, Japan). These mice were housed in a controlled environment with a 12:12-h light-dark cycle under standard laboratory chow and water. All experiments in this study were performed in accordance with the guidelines issued by the Institutional Animal Care and Use Committees of Hamamatsu University, School of Medicine, Japan (approval code: 2015028) and carried out in accordance with the approved guidelines.

### Sample preparation

Two mice were sacrificed by cervical dislocation, and immediately thereafter their kidneys were dissected and subsequently frozen in powdered dry ice. Stored organs at −80°C were sliced with a thickness of 10 μm using a cryostat microtome at −20°C (Leica CM1950, Leica Microsystems). The slices were mounted onto the indium tin oxide-coated glass slides (ITO glass, Matsunami Glass Ind., Ltd., Osaka, Japan) and stored again at −80°C in 50 mL Falcon tubes containing silica gel. Samples were coated with 9-aminoacridine (9AA; Merck Millipore, Darmstadt, Germany) solution at 10 mg/mL dissolved in 70% ethanol, as a matrix by an automatic sprayer (TM-Sprayer, HTX Technologies, North Carolina (NC), USA), soon before the MALDI MSI observation.

### MALDI-MSI

The MALDI-MSI observations of the kidney samples from the three mice were performed by iMScope (Shimadzu, Kyoto, Japan), as our previous study [28]. The best condition of the matrix and the mass range was determined, in which the signal from the samples is efficiently detected as a wide range of *m/z* as possible, and separated from the noise from the background and matrix. We set the condition as follows: with 9-AA matrix, mass spectra of the *m/z* range of 550–1050 were acquired under negative ion mode, with the scan pitch of 50 μm and a laser diameter of 25 μm. The laser strength was set to be 60%, and the number of irradiations was 500.

The negative ion mode was used with adduct type of [M-H]- and Molecular Mass Tolerance of ±0.2 Da. Lock mass correction was performed using *m/z* 885.5493 (for *m/z* >500) to achieve better mass accuracy [29] and candidate selection with higher precision [30]. *m/z* 885.5493 was annotated as phosphatidylinositol (PI)(38:4) [29].

### Shannon entropy calculation from MALDI-MSI data

Mass spectrum data was acquired by MALDI-MSI analysis. We used IMDX converter (Shimadzu Corporation, Kyoto, Japan) and IMAGEREVEAL^™^ MS software (Shimadzu Corporation, Kyoto, Japan) to get numerical data from the mass spectra. All peaks from the mass spectra were included for calculation. Let *P*_*i*_(*x*, *y*) denote the *i*th peak intensity in the mass spectrum at point (*x*, *y*) on the sample. We introduced the relative intensity as

$$p_{i}\left(x,y\right)=\frac{P_{i}\left(x,y\right)}{\sum_{i=1}^{n}P_{i}\left(x,y\right)},$$ (1)

where *n* is the number of peaks in the mass spectrum. Then we defined the Shannon entropy *H*(*x*, *y*) for the spot at (*x*, *y*) on the sample as

$$H\left(x,y\right)=-\sum_{i=1}^{n}p_{i}\left(x,y\right)\;{\text{log}}_{2}\;p_{i}\left(x,y\right).$$ (2)

The entropy heat map can be drawn with *H*(*x*, *y*). We note that the Shannon entropy depends on the number *n* of peaks (0 ≤ *H*(*x*, *y*) ≤ log_2_ *n*). The entropy *H*(*x*, *y*) reaches the maximum when all *p*_*i*_(*x*, *y*) are equiprobable. The Shannon entropy is high when the system contains much information and little certainty. The Shannon entropy is low when the number of possible values is small and the system is not random.

### Shannon entropy heat maps

**Fig 1:**
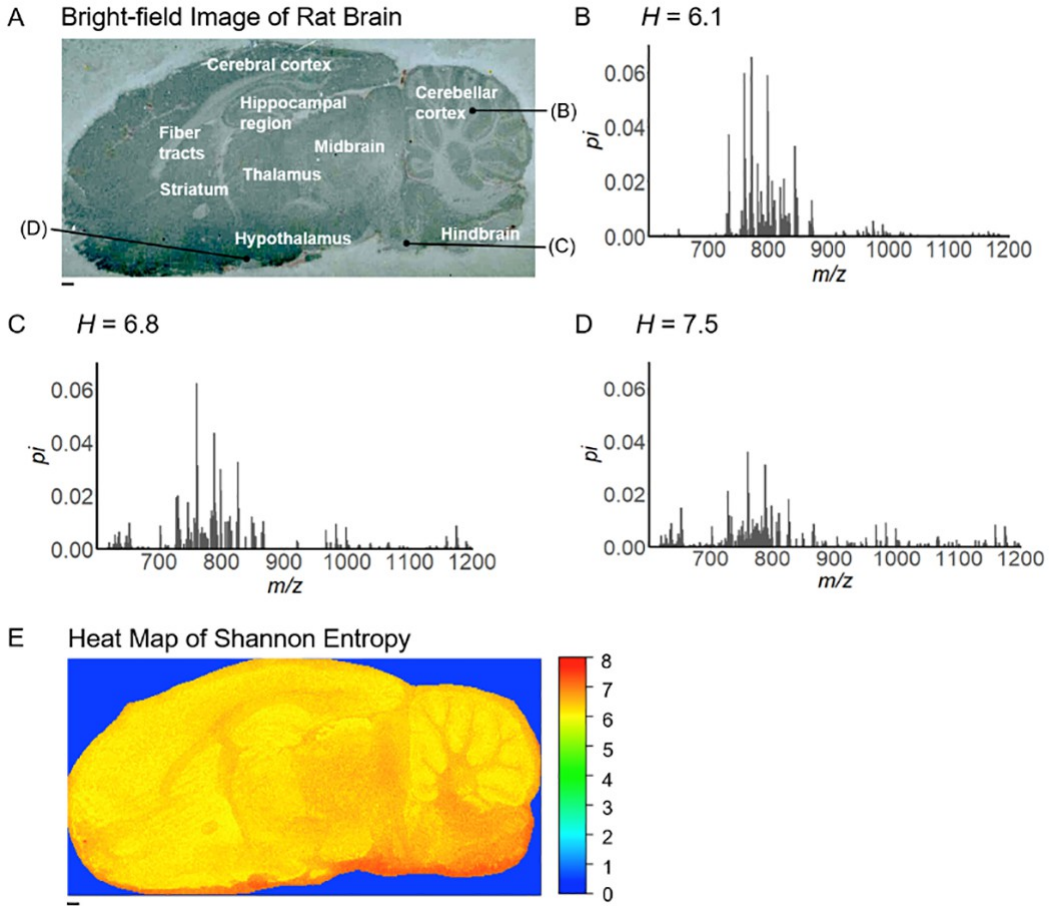
Shannon entropy calculated from the mass spectrum. (**A**) The bright-field image of the sagittal rat brain using MALDI-FT-ICR-MSI with 2,5-dihydroxybenzoic acid (DHB) matrix [20]. (**B-D**) Mass spectra at three different spots on the rat brain (*p*_*i*_: relative mass spectral intensity). At these spots, Shannon entropy values were 6.1 (**B**), 6.8 (**C**), and 7.5 (**D**), respectively. (**E**) The heat map of the Shannon entropy from the rat brain. The scale bar denotes 1 mm.

In the case of rat brain data, Wister male rats aged 8 weeks were used in the experiment. The MSI data of the rat brain data was previously obtained in our group by MALDI equipped with the Fourier transform ion cyclotron resonance mass spectrometry (FT-ICR) MSI (Solarix XR, Bruker Daltonics) with DHB matrix using iMLayer (Shimadzu Corporation, Kyoto, Japan) [31]. The bright-field image of the brain is shown in Fig 1A. Mass spectra at three different spots on the sample of the rat brain had different behavior (Fig 1B–1D). Upon the MSI measurement, the mass spectrum was acquired from each spot on the samples. We note that the Shannon entropy becomes high when the mass spectrum is uniformly distributed. Indeed, the Shannon entropy *H* for Fig 1B–1D was *H* = 6.1, 6.8, and 7.5, respectively.

Fig 1E shows the heat map of the Shannon entropy which is calculated from the mass spectrum at each spot. By comparing Fig 1A and 1E, we found that the entropy heat map reflected the anatomical structure. For instance, entropy values were relatively low (color close to yellow) in many parts of gray matter, including the cerebral cortex, hippocampal region, striatum, and cerebellar cortex. In contrast, entropy values are relatively high (color close to red) in many parts of the fiber tracts.

### Method to identify related peaks

With the help of entropy heat maps, we can find peaks related to a control parameter such as age. Our proposed method consists of the following four steps.

Step 1. Obtain entropy heat maps for different values of the control parameter and find a region in the heat maps which show a clear dependence on the control parameter. That is, a region of interest (ROI) is determined. Once an ROI is chosen for a heat map, ROIs must be set in the same area for other heat maps with different values of the control parameter.
Step 2. In the ROI, we calculate the sum of intensities for each peak in the spectrum. Sums are calculated for each value of the control parameter. Summed intensities are further normalized by using the sum from one value of the control parameter.
Step 3. The dependence of the normalized sums of intensities on the control parameter is investigated. Informative peaks are selected in the mass spectrum as peaks which significantly depend on the control parameter.
Step 4. Candidate molecules are identified from the selected peaks.

The workflow for our method to identify candidate molecules is illustrated in S1 Fig. Assignments of all candidate molecules were conducted with a good mass accuracy using the Human Metabolome Database (metabolite identification confidence level 3). The choice of the ROI in Step 1 is not unique. Depending on ROIs, different candidate peaks might be identified. Then it should be determined by the targeted MSI if the identified peaks are most important for the control parameter. The proposed scheme helps identify a few candidate peaks from hundreds of intensities on the *m/z* spectrum.

## Results

### Shannon entropy for the young and old kidneys

**Fig 2:**
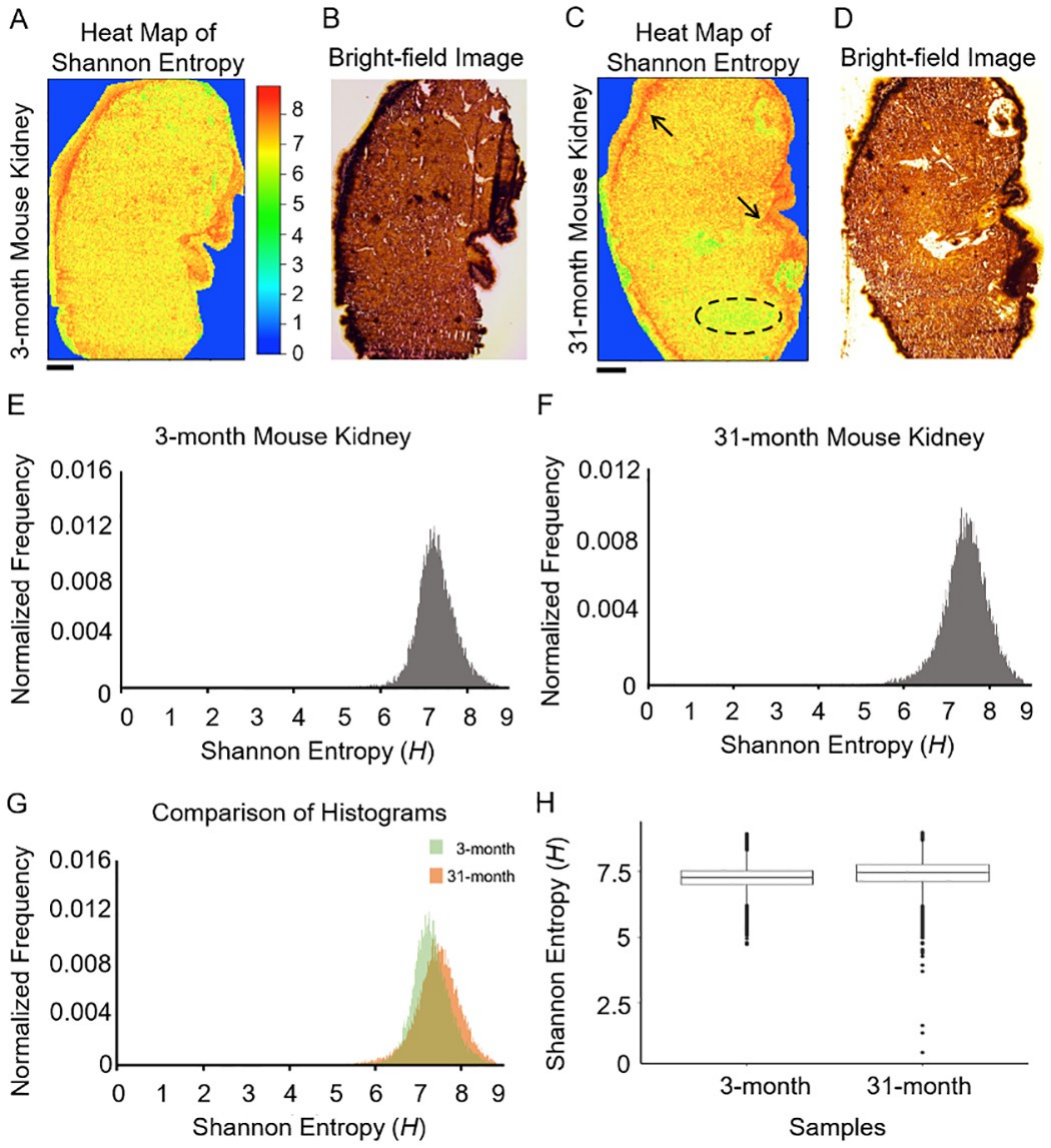
Shannon entropy for the two kidneys. Entropy heat maps (**A** and **C**) and bright-field images (**B** and **D**) are shown for the 3- and 31-month mouse kidneys, respectively. Black arrows show examples of high entropy regions. A low-entropy region which appeared only for the 31-month kidney was marked by a black dashed oval. (**E** and **F**) Histograms of the Shannon entropy of two samples. (**G**) The two histograms were superimposed. (**H**) Box plots for the 3- and 31-month kidneys. Scale bars denote 1 mm.

Shannon entropy heat maps for MSI were produced using young and old kidneys. The Shannon entropy was calculated from the MSI data acquired by MALDI equipped with the time-of-flight mass spectrometer (TOF-MS) for the kidneys of 3- and 31-month mice. Fig 2A and 2C shows Shannon entropy heat maps of these kidneys. For both kidneys, high entropy regions (marked in black arrows) were seen in the pelvis and the renal capsule. The number of low-entropy pixels increased for the 31-month kidney. For example, a low-entropy region (i.e., a group of low-entropy pixels) in the renal cortex (marked by a black dashed line) appeared. The histograms in Fig 2E and 2F show frequency distributions of the Shannon entropy for both kidneys. The histograms were normalized by the number of pixels. The average entropy of the 3-month kidney was smaller than that of the 31-month kidney (Fig 2G). The box plots of the entropy showed the existence of the low-value outliers in the 31-month kidney (Fig 2H).

### Low entropy spots for the young and old kidneys

**Fig 3:**
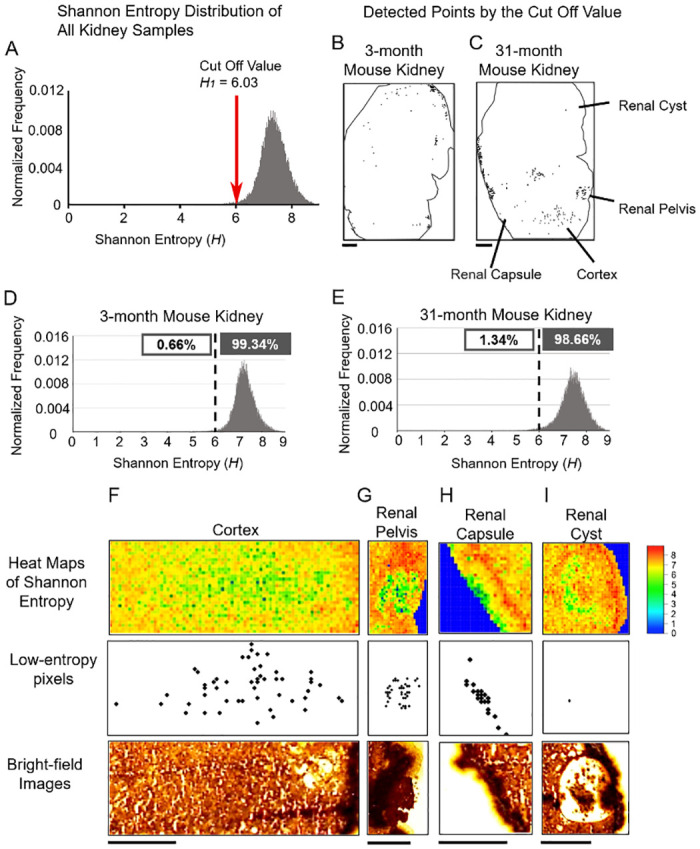
Detection of low-entropy spots in the mouse kidneys. (**A**) The distribution of the Shannon entropy from two kidney samples. (**B-C**) Low-entropy spots were plotted on the kidney images. Outlines of the kidneys are shown by black solid lines. (**D-E**) Histograms of the Shannon entropy for the 3- and 31-month mouse kidneys. Enlarged kidney images for the 31-month mouse of the cortex (**F**), pelvis (**G**), and the renal capsule (**H**) in addition to the renal cysts (**I**). Scale bars denote 1 mm.

The distribution of all entropy values of the samples was close to Gaussian (Fig 3A). To further investigate low-entropy pixels, we introduced a threshold *H*_1_. Among all pixels of the two samples, the smallest 1% of the pixels were defined as low entropy pixels (S1 Table) and correspondingly we set *H*_1_ = 6.03. When comparing the 3- and 31-month kidneys, we found more low-entropy spots in the 31-month kidney (Fig 3B and 3C). Indeed, 0.66% and 1.34% of pixels were classified as low-entropy pixels, respectively (Fig 3D and 3E). A magnified image of the 31-month kidney showed that even though the individual low-entropy spots were separated, as a group they exhibited a structure-like distribution in the cortex, renal pelvis, and renal capsule (Fig 3F–3H), while no low-entropy spots were found in the cyst with hollow structures (Fig 3I).

### Identification of candidate peaks using entropy heat maps

**Fig 4:**
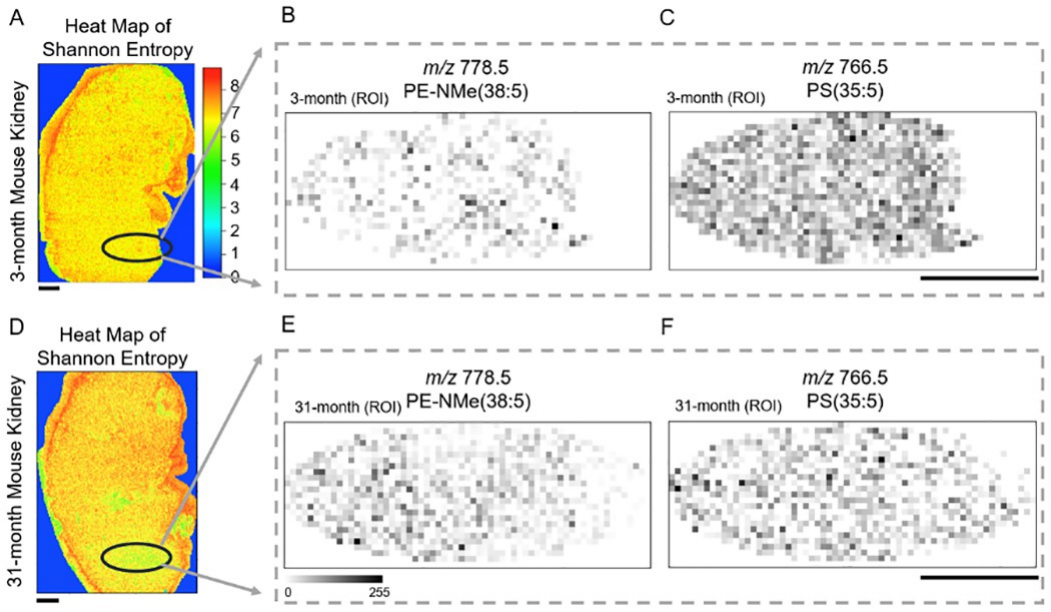
ROIs for two kidneys and MALDI-MSI ion images. (**A**) The entropy heat map for the 3-month kidney with the ROI marked by a black oval. (**B**) Ion image of *m/z* 778.5 for the 3-month mouse. (**C**) Ion image of *m/z* 766.5 for the 3-month mouse. (**D**) The entropy heat map for the 31-month kidney with the ROI marked by a black oval. (**E**) Ion image of *m/z* 778.5 for the 31-month mouse. (**F**) Ion image of *m/z* 766.5 for the 31-month mouse. Scale bars show 1 mm.

We first determined ROIs which contained low entropy regions. The ROIs are marked by black ovals in Fig 4A and 4D. For both samples, we calculated for each *m/z* the sum of the intensities for the pixels in ROIs. Then for each *m/z*, the ratio of the summed intensity for the 31-month kidney to the summed intensity for the 3-month kidney was calculated. From the list of change ratios, two *m/z*’s which corresponded to the largest and smallest ratios were picked (S2 Table).

In this way, two candidates were selected which had the largest and smallest change ratios. The peak of the largest change ratio (the ratio was 4.55) was *m/z* 778.5 and the peak of the smallest change ratio (the ratio was 0.380) was *m/z* 766.5. We note that by focusing on a particular intensity, more precise *m/z* values can be obtained: the former was *m/z* 778.5387 and the latter was *m/z* 766.4701.

The intensity with the largest change ratio (*m/z* 778.5387) was annotated as N-monomethylphosphatidylethanolamine (PE-NMe) (38:5). The intensity with the smallest change ratio (*m/z* 766.4701) was annotated as phosphatidylserine (PS)(35:5). Both candidate molecules were assigned according to the Human Metabolome Database (metabolite identification confidence level 3) (see Table 1). Furthermore, we produced MALDI-MSI ion images of the molecules in the ROIs for the 3-month kidney (Fig 4B and 4C) and 31-month kidney (Fig 4E and 4F). For *m/z* 778.5387, the average intensity for Fig 4B was smaller than that for Fig 4E. For *m/z* 766. 4701, the average intensity for Fig 4C was larger than that for Fig 4F.

**Table 1:** Tentative assignments of candidate molecules.

kidney					
Tentative ion attribution	Observed *m/z*	Database *m/z*	Mass error (ppm)	Adduct	Formula
PS(35:5)	766.4701	766.4665	4.70	[M-H]^-^	C_41_H_70_NO_10_P
PE-NMe(38:5)	778.5387	778.5392	0.64	[M-H]^-^	C_44_H_78_NO_8_P

### Robustness of low-entropy spots

**Fig 5:**
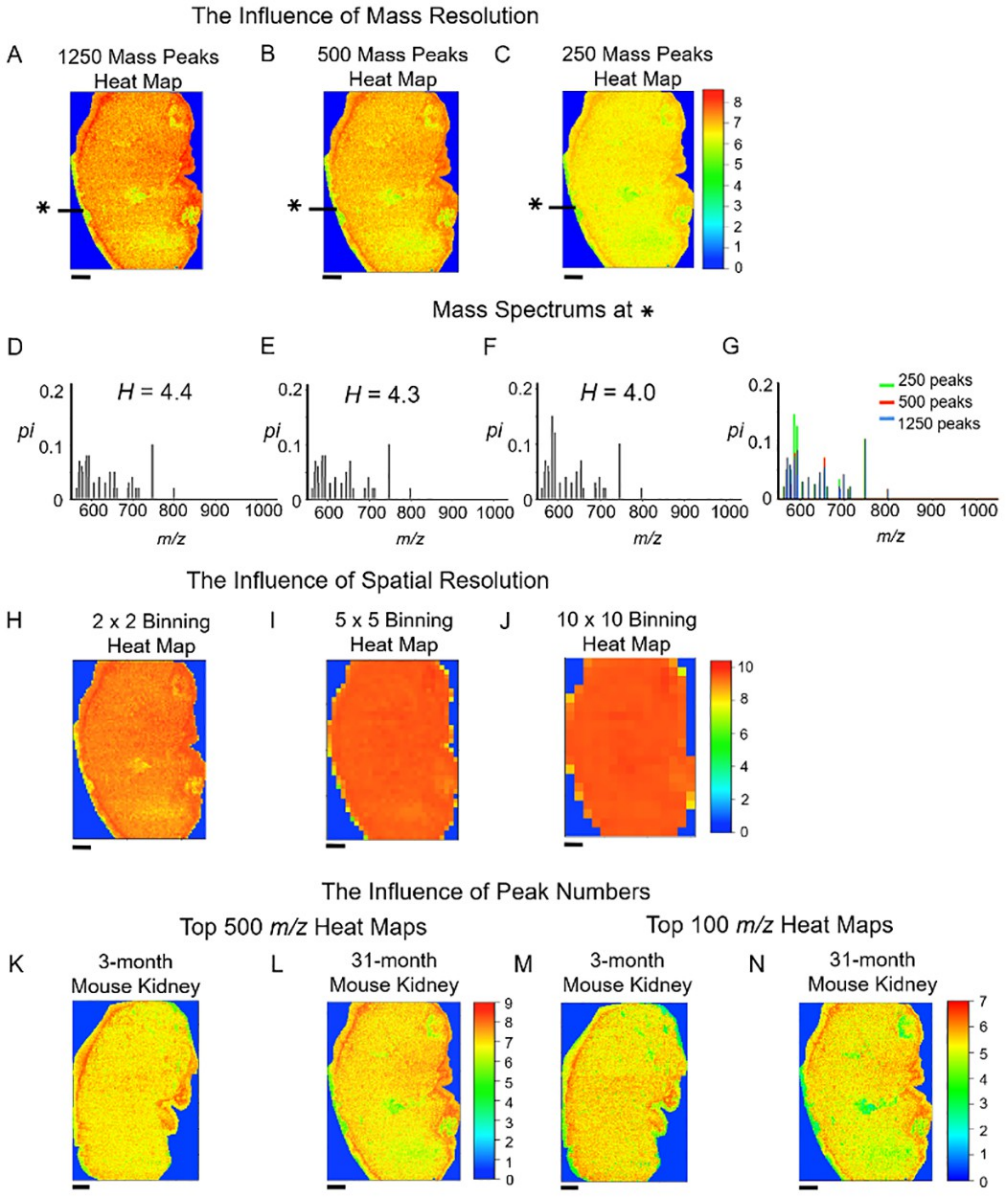
Effects of mass and spatial resolutions on entropy heat maps. (**A-C**) Entropy heat maps with low *m/z* resolutions created by the reduction of the number of intensities to (**A**) 1250, (**B**) 500, and (**C**) 250. (**D-F**) Mass spectra for three mass resolutions at the identical position (indicated * in (**A-C**)). (**G**) Three spectra together with the original spectrum with 2500 intensities. (**H-J**) Reduced spatial resolutions, by the binning of pixels by the factor (**H**) 2, (**I**) 5, and (**J**) 10. (**K-L**) Entropy heat maps with top 500 *m/z* peaks. (**M-N**) Entropy heat maps with top 100 *m/z* peaks. Scale bars indicate 1 mm.

To further investigate low-entropy spots, we changed mass and spatial resolutions for the 31-month mouse kidney. The mass resolution is commonly defined as the ability to separate two narrow mass spectral peaks. As the mass resolution gets lower, adjacent peaks become indistinguishable. At each spot, there were 2500 intensities in the *m/z*-axis as the interval from *m/z* 550 to *m/z* 1050 was split into 2500 subintervals. We took the average over every two, five, and ten neighboring intensities. Thus, we produced new heat maps with reduced numbers of peaks. As seen in Fig 5A–5C, newly obtained heat maps were more or less similar and the structure of low-entropy spots was preserved. High peaks appeared between *m/z* 550 and 800 for all mass spectra, which had 2500, 1250, 500, and 250 intensities (Fig 5D–5G). We found that key features such as low-entropy regions remain if the mass resolution changes.

To check the influence of the spatial resolution on the distribution of Shannon entropy, we reduced the spatial resolution by binning the MSI data, i.e., we took the average of mass spectra over adjacent spatial spots on the image. Unlike the case of the *m/z* resolution, which made no huge change in the distribution of the Shannon entropy, the reduction of the spatial resolution severely influenced the distribution (Fig 5H–5J). The entropy distribution became almost uniform after mass spectra in 25 (5 × 5) adjacent pixels in the image were averaged. Thus, high spatial resolution is necessary to detect entropy changes in the heat map.

To check the influence of peak selections on the distribution of the Shannon entropy, we summed all peaks from pixels in the sample and selected the top 500 and 100 *m/z* peaks among the summed peaks. With the top 500 and 100 *m/z* peaks, entropy heat maps were generated for the two kidney samples (Fig 5K–5N). The heat maps were more or less similar. This implies that the contribution of small peaks was negligible.

## Discussion

Entropy heat maps reflect the anatomical structure of the sample as shown in Fig 1. This is because entropy values vary depending on the anatomical structure. Moreover, entropy heat maps reveal features in the sample which cannot be detected by other modalities. An example of such features is the appearance of low-entropy regions in the kidney. Thus the Shannon-entropy heat map provides new insights for MSI. Although age was used as a control parameter in this study, different control parameters can be used for the proposed method.

We note that the information entropy carries the information from all peaks in the range of interest on the *m/z* axis. In this sense, the Shannon-entropy heat map is different from an image for a specific peak. Furthermore, the Shannon-entropy heat map differs from the total ion current or total ion image (sum of all ion intensities); the former is a weighted sum which measures the degree of randomness. See [26] for the comparison between Shannon entropy and total ion heat maps.

Since all peaks in the range which is considered are taken into account when the information entropy is calculated, matrix effects are unavoidable. We emphasize that as shown in this paper informative entropy heat maps can be obtained even in the presence of matrix.

We found that in the comparison between the two kidneys, entropy changes occurred in a small part of the cortex. This means that the spatial resolution must be below 100 μm. As written above, the laser diameter for the MSI measurements was set to 25 μm in this study.

In this paper, we identified two molecules, PS(35:5) (*m/z* 766.47) and PE-NMe(38:5) (*m/z* 778.54), to illustrate the proposed method of identifying relevant molecules via entropy heat maps. The results are consistent with the findings in our previous report [32]. In the present study, the 31-month kidney had more PE-NMe(38:5) than the 3-month kidney. PE treatment was reported to increase the averaged and maximum life span [33].

In Fig 5A–5C, the influence of the spectral binning on entropy heat maps was studied. The average entropy decreases as the bin size increases. In Fig 5A, the average entropy was 5.99 for the bin size 0.4 (1250 peaks). In Fig 5B, the average entropy was 5.76 for the bin size 1.0 (500 peaks). In Fig 5C, the average entropy was 5.41 for the bin size 2.0 (250 peaks). This tendency is implied in Fig 5D–5G. The result is consistent with [23], which investigated the spectral binning for TOF-SIMS data.

A natural next step is to perform more comprehensive experiments with the proposed analysis to identify molecules which are related to aging. MSI has been successfully applied in a wide range of kidney studies [34]. Among one of those studies, we previously developed the method to detect small metabolites in the kidney by MSI [35] and identified 6 specific lipids in immunoglobulin A (IgA) induced nephropathy in the mice model [36]. For the lipidomics analysis, Moreno-Gordaliza *et al*. determined the best condition for the broadest detection and identification of renal lipids by MALDI-MSI [37, 38]. They used 9-aminoacridine (9-AA) as a matrix for the negative ion-mode lipid imaging of the kidney, which is consistent with our condition for the kidney imaging. In the future research, it must be confirmed that PE-NMe(38:5) increases and PS(35:5) decreases as age grows.

Although in this paper we focused on the mass range between *m/z* 550 and *m/z* 1050, it is possible to consider the entire spectrum. Metabolic changes may be studied by entropy heat maps which are obtained from a wide range of the mass spectrum that contains small mass biomolecules such as lipids and peptides.

## Conclusions

In this paper, we have introduced entropy heat maps for MSI. We showed that entropy heat maps provide new information such as low-entropy regions, which cannot be imaged by other modalities. Furthermore, we proposed a method of detecting informative peaks in the mass spectrum from changes of entropy heat maps against a control parameter. Choosing age as a control parameter, we demonstrated how candidate molecules can be identified.

## Supporting information

S1 Fig Workflow of candidate molecules identification. After extraction of data from ROIs on each sample, summation and normalization are performed for intensities of each *m/z*. Then the fold-change is calculated to obtain *m/z* with higher degree of variations. Finally, tentative assignments of all candidate molecules are performed.
(TIF)

S2 Fig Pixels selected in the low entropy areas of different aged mice kidneys. Pixels selected in the low entropy of 3-month mouse kidney (**A**) and 31-month mouse kidney (**B**).
(TIF)

S1 Table Low-entropy spots by the cutoff at 6.03 for the 3-month kidney. (XLSX)

S2 Table Candidate peaks with change ratios (ascending order). (XLSX)
