# Supplementary figures and images for: Spatial distribution of the Shannon entropy for mass spectrometry imaging
Source: PLoS One. 2023 Apr 6;18(4):e0283966. doi: 10.1371/journal.pone.0283966 (PMC10079050; doi:10.1371/journal.pone.0283966)

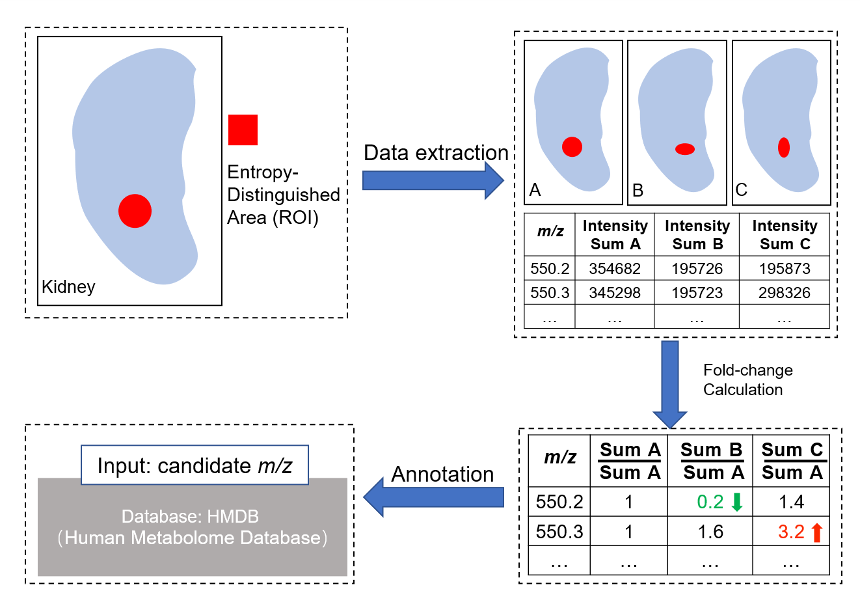

Supplement: S1 Fig — After extraction of data from ROIs on each sample, summation and normalization are performed for intensities of each m/z. Then the fold-change is calculated to obtain m/z with higher degree of variations. Finally, tentative assignments of all candidate molecules are performed. (TIF) [file pone.0283966.s001.tif]

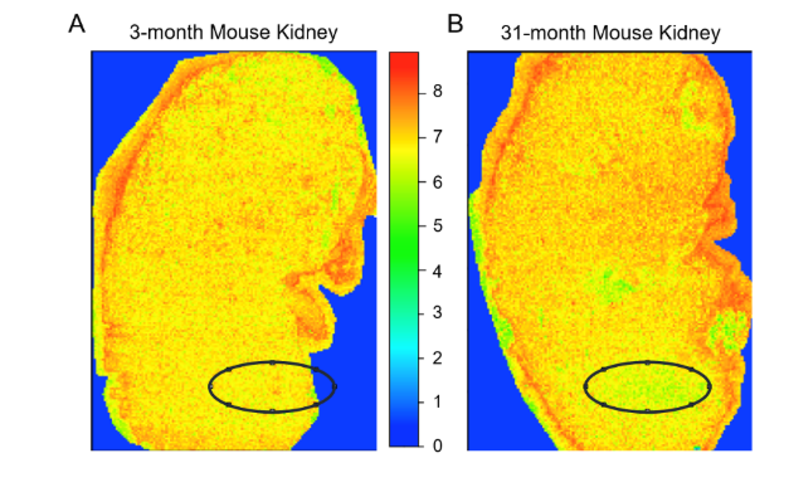

Supplement: S2 Fig — Pixels selected in the low entropy of 3-month mouse kidney (A) and 31-month mouse kidney (B). (TIF) [file pone.0283966.s002.tif]
